# Shen-Yuan-Dan Capsule Attenuates Verapamil-Induced Zebrafish Heart Failure and Exerts Antiapoptotic and Anti-Inflammatory Effects via Reactive Oxygen Species–Induced NF-κB Pathway

**DOI:** 10.3389/fphar.2021.626515

**Published:** 2021-03-01

**Authors:** Sinai Li, Hongxu Liu, Yue Li, Xiaomei Qin, Mengjie Li, Juju Shang, Wenlong Xing, Yanbing Gong, Weihong Liu, Mingxue Zhou

**Affiliations:** ^1^ Beijing Hospital of Traditional Chinese Medicine, Capital Medical University, Beijing Institute of Traditional Chinese Medicine, Beijing, China; ^2^ Beijing Hospital of Traditional Chinese Medicine, Capital Medical University, Beijing, China; ^3^ Dongzhimen Hospital, Beijing University of Chinese Medicine, Beijing, China

**Keywords:** heart failure, zebrafish, apoptosis, inflammation, NF-κB pathway, Shen-Yuan-Dan capsule

## Abstract

**Background:** Heart failure (HF) is the end stage of ischemic cardiovascular diseases; nonetheless, safe and effective therapeutic agents for HF are still lacking, and their discovery remains challenging. Our previous studies demonstrated that Shen-Yuan-Dan Capsule (SYDC), a hospital preparation of traditional Chinese herbal, effectively protected ischemic injury in cardiovascular diseases. However, its therapeutic effects and possible mechanisms on HF remain unclear.

**Methods**: A zebrafish HF model treated with verapamil was developed to assess the therapeutic effect of SYDC on HF zebrafish. Zebrafish were administered with SYDC and digoxin (positive control) by direct soaking. After drug treatment, zebrafish were randomly assigned to the visual observation and image acquisition using a Zebralab Blood Flow System. The reactive oxygen species (ROS), MDA, and SOD levels were determined by fluorescence signal detection, TBA, and WST-8 methods. RT-PCR determined the mRNA expressions of Caspase-3, Caspase-1, Bcl-2, Bax, IL-1β, NF-κB, and TNF-α.

**Results:** SYDC significantly inhibited the levels of heart dilatation and venous congestion and markedly increased the levels of cardiac output, blood flow dynamics, and heart rates in HF zebrafish (*p* < 0.05, *p* < 0.01, and *p* < 0.001). Moreover, SYDC also significantly decreased the levels of MDA and ROS and increased the level of SOD in HF zebrafish. The RT-PCR results revealed that SYDC decreased the expression of Caspase-1, Caspase-3, Bax, IL-1β, NF-κB, and TNF-α but increased the expression of Bcl-2 in HF zebrafish (*p* < 0.05, *p* < 0.01, and *p* < 0.001).

**Conclusions:** SYDC improved the heart function in verapamil-induced HF zebrafish and alleviated inflammation and apoptosis by inhibiting the ROS-mediated NF-κB pathway.

## Introduction

Heart failure (HF) is an important public health problem that affects more than 23 million people worldwide (Marios et al., 2020). It is a highly prevalent disease that constitutes a major medical and economic burden in the healthcare system (Lesyuk et al., 2018). HF is the end stage of ischemic cardiovascular disease with clinicopathological characteristics that include cardiac enlargement, severe venous congestion, insufficient cardiac output, slow heartbeat, and blood flow rate (Zolt and Uri, 2016). Despite improvements in medical treatment, the prognosis in patients with HF remains poor (Mosterd and Hoes, 2007). Therefore, the discovery of safe and effective therapeutic agents for HF is challenging.

Conventional mammalian HF models are usually laborious, costly, time-consuming, and restricting their applications in pharmacology (Delgad, 2004). Over recent years, zebrafish have been used as a novel cardiovascular disease animal model with good visibility for assessing drug toxicity, efficacy, and drug screening (Huang et al., 2013; Kim et al., 2013; Shi et al., 2017). Zhu *et al.* reported that the verapamil-treated larval zebrafish was convenient and predictive for rapid *in vivo* efficacy assessment and screening of HF therapeutic drugs (Zhu et al., 2018).

Oxidative stress has a vital role in the occurrence and development of HF (Tsutsui et al., 2011). In patients with HF and *in vivo* models, excessive oxidative stress induced by reactive oxygen species (ROS) production in the myocardium is accompanied by systemic inflammation and apoptosis (Ambrish et al., 2019). ROS-mediated nuclear factor-kappa B (NF-κB) signaling pathway is an emerging inflammatory and apoptosis signaling pathway (Sahar et al., 2020). Accumulation of ROS leads to the activation of NF-κB, which is the basic transcription factor that activates inflammation (Mieczysław et al., 2020). Prolonged activation of NF-κB causes the release of both a large number of proinflammatory cytokines and an intensification of cardiomyocyte apoptosis (Mieczysław et al., 2020).

Shen-Yuan-Dan capsule (SYDC), a hospital preparation of traditional Chinese herbal medicine, has been used for clinical treatment of unstable angina pectoris for more than 30 years with favorable effect (Li et al., 2019). Previous studies have shown that it can inhibit oxygen-free radicals in ischemic myocardium, improve myocardial tissue's antioxidant ability, and inhibit inflammatory reaction *in vivo* (Liu et al., 2013; Zhou et al., 2017). According to the crucial role of oxidative stress-inducing apoptosis and systemic inflammation in HF development and our previous studies on SYDC preventing unstable angina pectoris through its antioxidative effects, we hypothesized that SYDC could attenuate HF by inhibiting the oxidative stress and the activation of apoptosis and inflammation. Therefore, this study aimed to explore the protective role of SYDC against HF by establishing the verapamil-induced larval zebrafish HF model and investigating the underlying mechanism of this protective effect.

## Materials and Methods

### Zebrafish Care and Maintenance

Adult AB strain zebrafish were housed in a light- and temperature-controlled aquaculture facility with a standard 14 h light/10 h dark photoperiod and fed with brine shrimp twice daily and dry flake once a day. Four to five pairs of zebrafish were set up for natural mating every time. On average, 200–300 embryos were generated. Embryos were maintained at 28°C in fish water (0.2% instant ocean salt in deionized water, pH 6.9–7.2, conductivity 480–510 μS/cm, and hardness 53.7–71.6 mg/L CaCO_3_) (Zhou et al., 2015). The embryos were cleaned and staged at 2 dpf (days post fertilization). The zebrafish facility at Hunter Biotechnology, Inc. is accredited by the Association for Assessment and Accreditation of Laboratory Animal Care (AAALAC) International. The zebrafish were purchased from Hunter Biotechnology Co., Ltd. (Hangzhou, China).

### Ethics Approval

All animal research conformed to the Guidelines for the Care and Use of Laboratory Animals published by the US National Institutes of Health and was approved by the Ethics Review Board for Animal Studies of Peking University Health Science Center (permit number: IMM-GuYC-6).

### Drugs and Collocation

Verapamil (lot #: K1417048) and digoxin (lot #: L1303078) were purchased from Aladdin Chemical Co., Ltd. (Shanghai, China). Ultrapure water was used to prepare the mother liquor of verapamil with a concentration of 20 mM. The concentration of verapamil to induce HF in zebrafish at 2 dpf was 200 mM. Dimethyl sulfoxide (DMSO) was used to prepare the mother liquor of digoxin with a concentration of 8 mg/ml. SYDC was provided by Beijing TCM Hospital (Beijing, China, Z20053327), and the mother liquor of 20 mg/ml was prepared with normal saline before the experiment. SYDC is composed of eight traditional herbs: *Hirudo*, *Astragali Radix*, *Codonopsis Radix*, *Pheretima*, *Eupolyphaga Seu Steleophaga*, *Corydalis Rhizoma*, *Salviae Miltiorrhizae Radix et Rhizoma*, and *Scrophulariae Radix.* The corresponding ratio of the plants present in SYDC is 3:6:4:3:3:3:4:3.

### HF Zebrafish Model Development

Zebrafish at 2 dpf were chosen as an appropriate stage to start verapamil treatment to establish the HF model. Zebrafish at 2 dpf were treated with 200 mM verapamil for 30 min so as to induce HF (Huang et al., 2013). After verapamil treatment, ten zebrafish from each group were randomly assigned to the visual observation; the images were acquired at the diastolic stage of zebrafish heart beating without anesthetic by a dissecting stereomicroscope (Olympus, Japan). Quantitative image analysis was performed using image-based morphometric measurement. The other zebrafish were subjected to video recording using a Zebralab Blood Flow System (Viewpoint, France). Quantitative analysis was performed using video-based measurement. Qualitative and quantitative results of area measurements of heart dilatation and venous congestion, cardiac output, and blood flow dynamics reduction were used to assess whether the HF zebrafish model was established successfully (Zhu et al., 2018).

### Determination of No Observed Adverse Effect Level

To determine the no observed adverse effect level (NOAEL) of SYDC, zebrafish at 2 dpf were administered with SYDC for 4 h, and mortality and toxicity were recorded during the treatment. Five concentrations (250 μg/ml, 500 μg/ml, 1000 μg/ml, 1500 μg/ml, and 2000 μg/ml for soaking drugs) were used for SYDC. The NOAEL of SYDC was settled as the maximum concentration or maximum dose that did not lead to any observable side effect in zebrafish. This was determined under a dissecting stereomicroscope by a well-trained zebrafish toxicologist (Westerfield, 1999).

### Assessment Effects of SYDC on HF Zebrafish

To assess the effects of the SYDC on HF zebrafish, thirty AB strain zebrafish at 2 dpf were randomly assigned into six-well plates in 3 ml of fish water. Zebrafish were pretreated with SYDC for 4 h at serial concentrations followed by treatment with 200 mM verapamil for 30 min (Zhu et al., 2018). Zebrafish treated with 0.1% DMSO or 0.9% sodium chloride were used as vehicle controls. Untreated zebrafish were used to confirm that the vehicle solvent did not induce any side effects in zebrafish. Heart dilatation and venous congestion in zebrafish were visually tracked and confirmed under a dissecting stereomicroscope. The cardiac output and blood flow dynamics in zebrafish were recorded using a Zebralab Blood Flow System.

The improved efficiency of SYDC on heart dilatation and venous congestion was calculated using the following formula: *treatment efficiency* = (*the model control group* – *SYDC treatment group*)*/*(*the model control group* – *the normal control group*). The improved efficiency of SYDC on cardiac output, heart rate, and blood flow dynamics was calculated as follows: *treatment efficiency* = (*SYDC treatment group* – *the model control group*)*/*(*the normal control group* – *the model control group*) (Zhu et al., 2018).

### Determination of MDA Levels of HF Zebrafish

Four hundred eighty wild-type AB strain zebrafish were randomly selected in six-well plates three days after fertilization (3 dpf), and 30 zebrafish were treated in each hole (experimental group). The experimental group was treated with water-soluble SYDC (2000 ug/mL) and digoxin (0.1 ug/mL). The normal control group (zebrafish treated with water for fish farming) and the model control group were set up at the same time. The capacity of each hole (experimental group) was 3 ml. Four groups were set up in parallel in each experimental group. After being treated with SYDC and digoxin for 4 h, all the experimental groups, except the normal control group, were treated with verapamil to induce HF in zebrafish. At the end of the experiment, samples were taken according to the TBA method (lipid oxidation MDA detection kit, Biyun Tian), and the content was determined to evaluate the effects of SYDC on the level of MDA in HF zebrafish.

### Determination of ROS Levels of HF Zebrafish

One hundred twenty wild-type AB strain zebrafish were randomly selected 3 days after fertilization (3 dpf) and treated in a six-well plate with 30 zebrafish in each hole (experimental group). Water-soluble SYDC (2000 ug/mL) and digoxin (0.1 ug/mL) were treated, respectively. The normal control group (zebrafish treated with water for fish farming) and the model control group were set up at the same time. The capacity of each hole (experimental group) was 3 ml. After being treated with SYDC and digoxin for 4 h, all the experimental groups, except the normal control group, were treated with verapamil to induce HF zebrafish. At the end of the experiment, ROS detection reagent was added to each group, after which zebrafish were transferred to a 96-well plate, one tail per hole (10 holes per group) with a capacity of 100 L per hole. The 96-well plate was placed in the incubator at 28°C for 20 h, and the fluorescence signal intensity of zebrafish was read by a multifunction enzyme marker. The results were analyzed based on fluorescence signal intensity.

### RT-PCR

The total RNA of mice tissues was extracted using a TRIzol kit according to the manufacturer’s instructions. The primers of Caspase-3, Caspase-1, Bcl-2, Bax, IL-1β, NF-κB, TNF-α, and beta-actin are shown in Table 1. The protocol for RT-PCR was according to our previous method (Zhang et al., 2017).

**TABLE 1:** Primer sequence information in this study.

Genes	Primer sequence (5′→3′)	
β-Actin	Forward	TCG​AGC​AGG​AGA​TGG​GAA​CC
	Reverse	CTC​GTG​GAT​ACC​GCA​AGA​TTC
IL-1β	Forward	GTC​ACA​CTG​AGA​GCC​GGA​AG
	Reverse	GCA​GGC​CAG​GTA​CAG​GTT​AC
Bcl-2	Forward	CAC​TGG​ATG​ACT​GAC​TAC​CTG​AA
	Reverse	CCT​GCG​AGT​CCT​CAT​TCT​GTA​T
Bax	Forward	GAC​TTG​GGA​GCT​GCA​CTT​CT
	Reverse	TCC​GAT​CTG​CTG​CAA​ACA​CT
NF-κB	Forward	GATGTTCACTGCGTTCCT
	Reverse	GTC​TTC​TGT​CTC​TTC​CTC​TG
TNF-α	Forward	ATC​TTC​AAA​GTC​GGG​TGT​ATG
	Reverse	TGTGCCCAGTCTGTCTCC
Caspase-1	Forward	GTG​GTC​ACC​GAA​TGC​CAG​TA
	Reverse	TCG​CAG​CAA​GGT​TTT​CCT​CT
Caspase-3	Forward	TGT​GAC​CAC​TGG​CAT​TCA​TT
	Reverse	TCC​TCC​CTC​TTC​CGA​GAT​TT

### Statistical Analyses

One-way ANOVA, followed by Dunnett’s *t*-test, was used to compare differences among groups. All statistical analyses were performed using the SPSS 16.0 software (SPSS, United States). The quantitative data were presented as mean ± SEM. All experiments were repeated at least three times. *P*-value of <0.05 was considered statistically significant.

## Results

### SYDC Composition

We used UPLC-MS/MS to determine the composition of SYDC. As shown in Supplementary Figure S1, the total ion chromatograms of SYDC included tetrahydropalmatine, harpagoside, salvianolic acid A, salvianolic acid B, and tanshinone IIA. The mass spectrograms and chemical formulas of the main ingredients of SYDC are shown in Supplementary Figures S2 and S3.

### Determination of NOAEL in SYDC-Treated HF Zebrafish

Cardiovascular severe toxicity, including pericardial edema, bradycardia, or no blood circulation, was observed in all HF zebrafish treated with SYDC at 250 μg/ml, 500 μg/ml, 1000 μg/ml, 1,500 μg/ml, and 2000 μg/ml. No observable toxicity or death was found in HF zebrafish after SYDC treatment at a series of concentrations (Table 2). As our results showed, it was safe to treat HF zebrafish below 2,000 μg/ml 222 μg/ml, 666 μg/ml, and 2,000 μg/ml SYDC which were chosen as SYDC-low dose (SYDC-L), SYDC-middle dose (SYDC-M), and SYDC-high dose (SYDC-H) to investigate the dose-effect relationship between SYDC and HF.

**TABLE 2:** The statistic on the number of Zebrafish deaths at the detection concentration of SYDC (*n* = 30).

Group	Concentration	Zebrafish reaction	Number of deaths (tail)	Mortality (%)	Maximum drug concentration
Normal control					
Model control			0	0	
Solvent control group			0	0	
SYDC	250 μg/ml	No visible abnormality	0	0	2000 μg/ml
	500 μg/ml	No visible abnormality	0	0	
	1,000 μg/ml	No visible abnormality	0	0	
	1,500 μg/ml	No visible abnormality	0	0	
	2000 μg/ml	No visible abnormality	0	0	

### Verapamil Induces HF in Zebrafish

**FIGURE 1:**
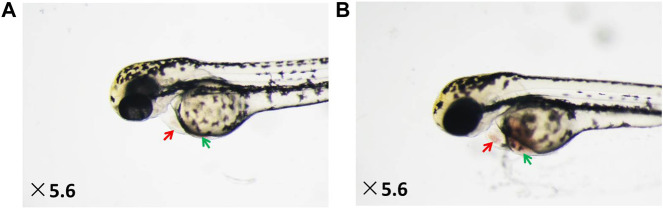
Heart dilatation and venous congestion in HF zebrafish treated with verapamil. **(A)** Vehicle-treated zebrafish (normal control group); **(B)** zebrafish treated with verapamil (model control group).

**FIGURE 2:**
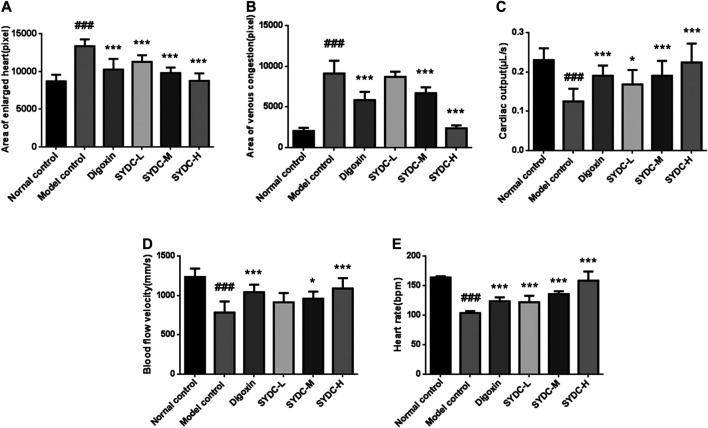
The dose-dependent improvement of enlarged heart area, venous congestion, cardiac output, blood flow velocity, and heart rates in HF zebrafish treated with SYDC for 4 h. **(A)** The heart areas of the zebrafish in each group. **(B)** The venous congestion areas of the zebrafish in each group. **(C)** The cardiac output of the zebrafish in each group. **(D)** The blood flow velocity of the zebrafish in each group. **(E)** The heart rates of the zebrafish in each group. ^###^Compared with the normal control, *p* < 0.001; *compared with the model control, *p* < 0.05; ***compared with the model control, *p* < 0.001.

As shown in Figure 1, under a dissecting stereomicroscope, the hearts of zebrafish after verapamil treatment were obviously enlarged (as shown by red arrows), and obvious venous congestion developed (as shown by the green arrow) compared with those of zebrafish in the normal control group. According to the qualitative and quantitative image-based morphometric analyses, the areas of an enlarged heart and venous congestion of the zebrafish in the model control group were significantly increased compared with those of zebrafish in the normal control group (13,344 ± 279.03 pixels *versus* 8,683 ± 272.12 pixels; 9,097 ± 499.04 pixels *versus* 2035 ± 120.13 pixels, *p* < 0.001). Moreover, the cardiac outputs, blood flow velocities, and heart rates of the zebrafish in the model control group were significantly decreased compared with those of the zebrafish in the normal control group (0.12 ± 0.01 ULL/s *versus* 0.23 ± 0.01 ULL/s; 784 ± 44 mm/s *versus* 1,235 ± 34 mm/s; 104 ± 1.02 times/min *versus* 164 ± 0.66 times/min, *p* < 0.001) (Figure 2). The above data suggest that the HF zebrafish model was successfully established.

### SYDC Reduces the Areas of Enlarged Heart in HF Zebrafish

**FIGURE 3:**
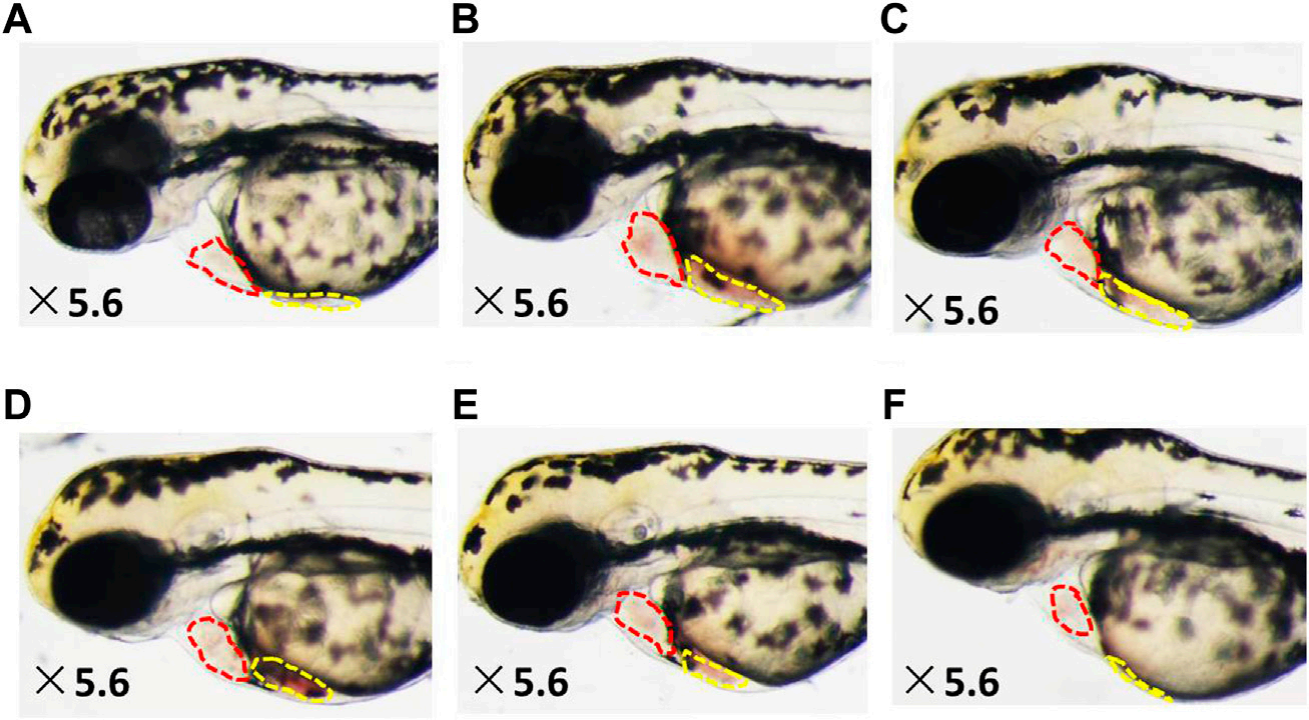
Reduced heart dilatation and venous congestion in HF zebrafish treated with SYDC for 4 h. **(A)** Vehicle-treated zebrafish; **(B)** zebrafish treated with verapamil alone; and HF zebrafish treated with digoxin, SYDC-low dose, SYDC-middle dose, and SYDC-high dose, resulting in significantly decreased heart dilatation and venous congestion **(C–F)**. The red-dotted lines indicate the heart area of zebrafish; the yellow-dotted lines indicate the venous congestion area of zebrafish.

**FIGURE 4:**
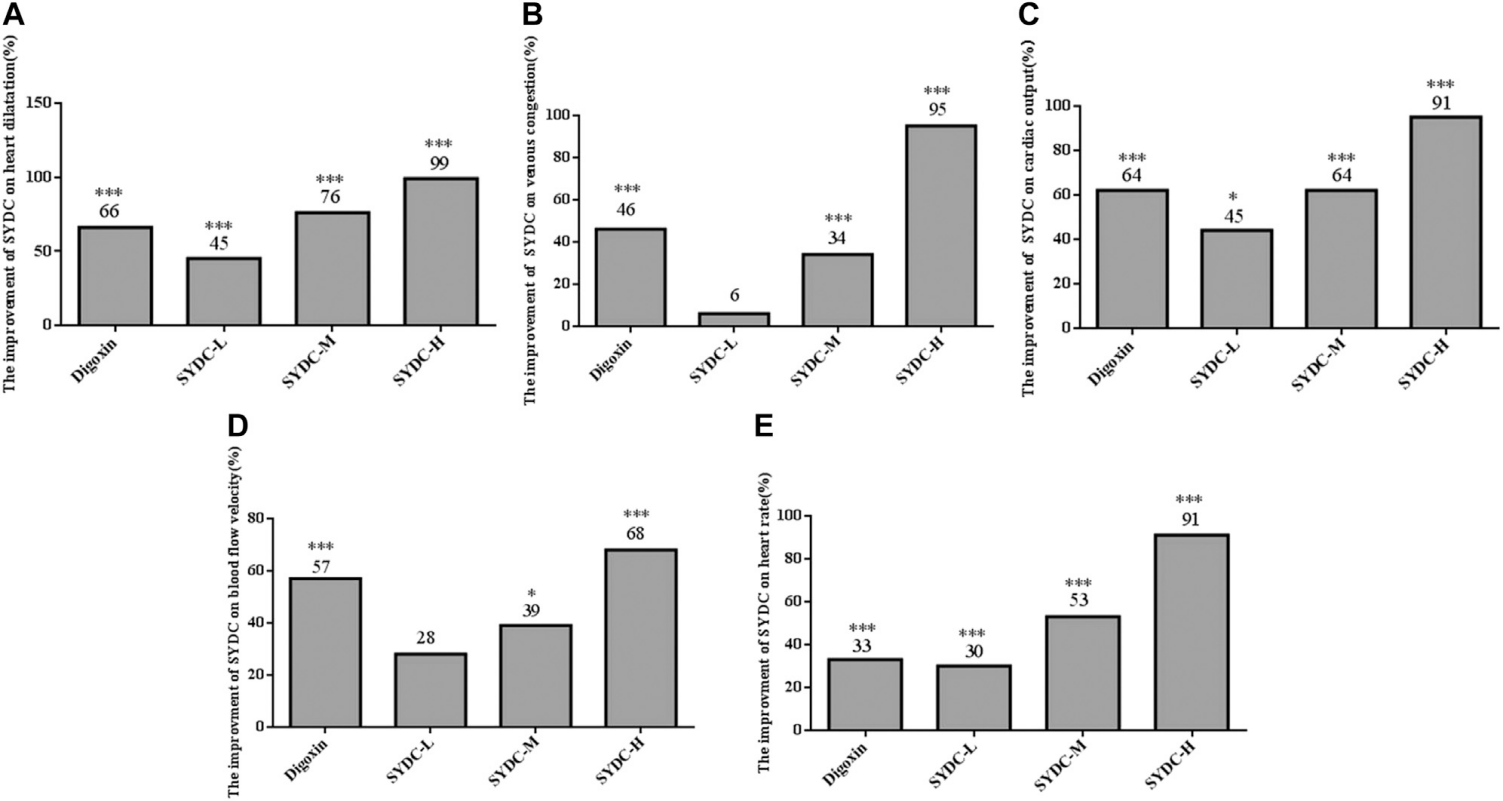
The efficiency improvement of enlarged heart area, venous congestion, cardiac output, blood flow velocity, and heart rates in HF zebrafish treated with SYDC for 4 h. *Compared with the model control, *p* < 0.05; ***compared with the model control, *p* < 0.001.

As shown in Figures 2–4, the areas of enlarged hearts of the HF zebrafish in the SYDC-L, SYDC-M, and SYDC-H treatment groups were significantly decreased compared with those of HF zebrafish in the model control group (11,254 ± 279 pixels, 9,781 ± 230 pixels, and 8,750 ± 314 pixels *versus* 13,344 ± 279 pixels, *p* < 0.001). The reduction of cardiac enlargement in the SYDC-L, SYDC-M, and SYDC-H treatment groups was 45%, 76%, and 99%, respectively, compared with that of HF zebrafish in the model control group (*p* < 0.001). The areas of enlarged hearts of the zebrafish in the digoxin treatment group were significantly decreased compared with those of HF zebrafish in the model control group (10,249 ± 447 pixels *versus* 13,344 ± 279 pixels, *p* < 0.001). The reduction of cardiac enlargement in HF zebrafish in the digoxin treatment group was 66% compared with that of HF zebrafish in the model control group (*p* < 0.001).

### SYDC Reduces Areas of Venous Congestion of HF Zebrafish

As shown in Figures 2–4, the areas of venous congestion of the HF zebrafish in the SYDC-M and SYDC-H treatment groups were significantly decreased compared with those of HF zebrafish in the model control group (6,685 ± 233 and 2,372 ± 111 *versus* 9,097 ± 499 pixels, *p* < 0.001), while the areas of venous congestion of the HF zebrafish in the SYDC-L treatment group were not significantly changed compared with those of HF zebrafish in the model control group (*p* > 0.05). The reduction of venous congestion of HF zebrafish in the SYDC-M and SYDC-H treatment groups was 34% and 95%, respectively, compared with that of HF zebrafish in the model control group (*p* < 0.001). The areas of venous congestion of HF zebrafish in the digoxin treatment group were significantly decreased compared with those of HF zebrafish in the model control group (5,835 ± 322 pixels *versus* 9,097 ± 499 pixels, *p* < 0.001). The reduction of venous congestion of the HF zebrafish in the digoxin treatment group was 46% compared with that of the HF zebrafish in the model control group (*p* < 0.001).

### SYDC Increases Cardiac Output of HF Zebrafish

As shown in Figures 2 and 4, the cardiac output of HF zebrafish in the SYDC-L, SYDC-M, and SYDC-H treatment groups was significantly increased compared with that of HF zebrafish in the model control group (0.17 ± 0.01 μL/s, 0.19 ± 0.01 μL/s, and 0.22 ± 0.02 μL/s *versus* 0.12 ± 0.01 μL/s, *p* < 0.05; *p* < 0.001). The increase of cardiac output of HF zebrafish in the SYDC-L, SYDC-M, and SYDC-H treatment groups was 45%, 64%, and 91%, respectively, compared with that of HF zebrafish in the model control group (*p* < 0.001). The cardiac output in HF zebrafish in the digoxin treatment group was significantly increased compared with that of HF zebrafish in the model control group (0.19 ± 0.01 μL/s *versus* 0.12 ± 0.01 μL/s, *p* < 0.001). The increase of cardiac output in HF zebrafish in the digoxin treatment group was 64% compared with that of HF zebrafish in the model control group (*p* < 0.001).

### SYDC Increases Blood Flow Velocity of HF Zebrafish

As shown in Figures 2 and 4, the blood flow velocities of HF zebrafish in the SYDC-M and SYDC-H treatment groups were significantly increased compared with those of HF zebrafish in the model control group (912 ± 37 mm/s, 959 ± 28 mm/s, and 1,088 ± 41 mm/s *versus* 784 ± 44 mm/s, *p* < 0.001), while the blood flow velocities of the HF zebrafish in the SYDC-L treatment group were not significantly changed compared with those of HF zebrafish in the model control group (*p* > 0.05). The increase of blood flow velocity in HF zebrafish in the SYDC-M and SYDC-H treatment groups was 39% and 68%, respectively (*p* < 0.001). The blood flow velocities of the HF zebrafish in the digoxin treatment group were significantly increased compared with those of HF zebrafish in the model control group (1,041 ± 30 mm/s *versus* 784 ± 44 mm/s, *p* < 0.001). The increase of blood flow velocities in HF zebrafish in the digoxin treatment group was 57% compared with that of HF zebrafish in the model control group (*p* < 0.001).

### SYDC Increases Heart Rate in HF Zebrafish

The heart rates of HF zebrafish in the SYDC-L, SYDC-M, and SYDC-H treatment groups were significantly increased compared with those of HF zebrafish in the model control group (122 ± 3.47 times/min, 136 ± 1.47 times/min, and 158 ± 4.85 times/min *versus* 104 ± 1.02 times/min, *p* < 0.001) (Figures 2 and 4). The increase of heart rate in HF zebrafish in the SYDC-L, SYDC-M, and SYDC-H treatment groups was 30%, 53%, and 91%, respectively (*p* < 0.001). The heart rates of the zebrafish in the digoxin treatment group were significantly increased compared with those of HF zebrafish in the model control group (124 ± 2.04 mm/s *versus* 104 ± 1.02 mm/s, *p* < 0.001). The increase of heart rates in HF zebrafish in the digoxin treatment group was 33% compared with that of HF zebrafish in the model control group (*p* < 0.001). The above data suggest that SYDC can dose-dependently diminish the HF zebrafish.

### SYDC Inhibits Oxidative Stress in HF Zebrafish

**FIGURE 5:**
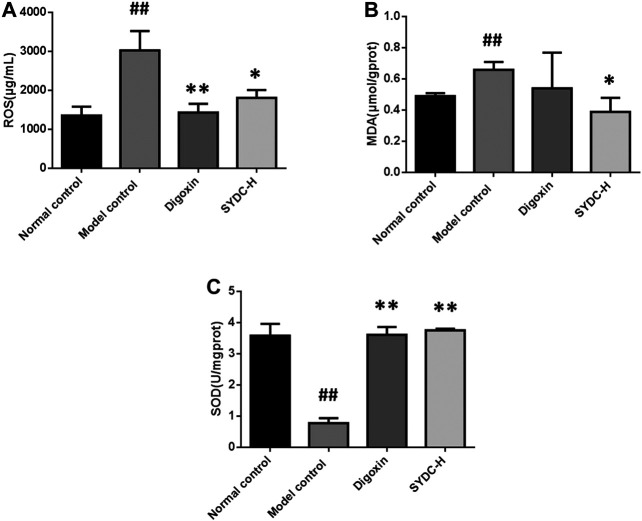
The ROS, MDA, and SOD levels of HF zebrafish treated with SYDC for 4 h. ^##^,Compared with the normal control, *p* < 0.01; *compared with the model control, *p* < 0.05, **compared with the model control, *p* < 0.01.

Oxidative stress induced by ROS has a vital role in the development of HF (Tsutsui et al., 2011). To a certain extent, the levels of MDA and SOD can reflect the degree of oxidative stress in HF (Anna et al., 2020). Because the curative effect of SYDC-H treatment groups on HF zebrafish was markedly superior to the other dose treatment groups in the present study, we only chose the SYDC-H treatment group to investigate the mechanism of SYDC on HF zebrafish. As shown in Figure 5, the activities of ROS and MDA levels of the HF zebrafish in the model control group were significantly increased compared with those of the HF zebrafish in the normal control group (3033 ± 493.90 μmol/gprot *versus* 1,358 ± 225.60 μmol/gprot, *p* < 0.01; 0.66 ± 0.05 μmol/gprot *versus* 0.4 ± 0.02 μmol/gprot, *p* < 0.05), while the SOD levels of the HF zebrafish in the model control group were significantly decreased compared with those of the HF zebrafish in the normal control group (0.78 ± 0.16 U/mgprot *versus* 3.59 ± 0.38 U/mgprot). The activities of ROS in HF zebrafish in the SYDC-H treatment group and digoxin treatment group were significantly decreased compared with those of HF zebrafish in the model control group (1810 ± 200.50 μmol/gprot *versus* 3033 ± 493.90 μmol/gprot, *p* < 0.05; 1,436 ± 221.50 μmol/gprot *versus* 3033 ± 493.90 μmol/gprot, *p* < 0.01). The levels of MDA of HF zebrafish in the SYDC-H treatment group were significantly decreased compared with those of HF zebrafish in the model control group (0.39 ± 0.09 μmol/gprot *versus* 0.66 ± 0.05 μmol/gprot, *p* < 0.05). The levels of MDA of HF zebrafish in the digoxin treatment group did not significantly change compared with those of HF zebrafish in the model control group (*p* > 0.05). The SOD levels of the HF zebrafish in the SYDC-H treatment group and the digoxin treatment group were significantly increased compared with those of HF zebrafish in the model control group (3.76 ± 0.05 U/mgprot *versus* 0.78 ± 0.16 U/mgprot, *p* < 0.01; 3.62 ± 0.25 U/mgprot *versus* 0.78 ± 0.16 U/mgprot, *p* < 0.01). There were no significant differences between the two drug treatment groups (*p* > 0.05).

### SYDC Inhibits Inflammatory Factors in HF Zebrafish

**FIGURE 6:**
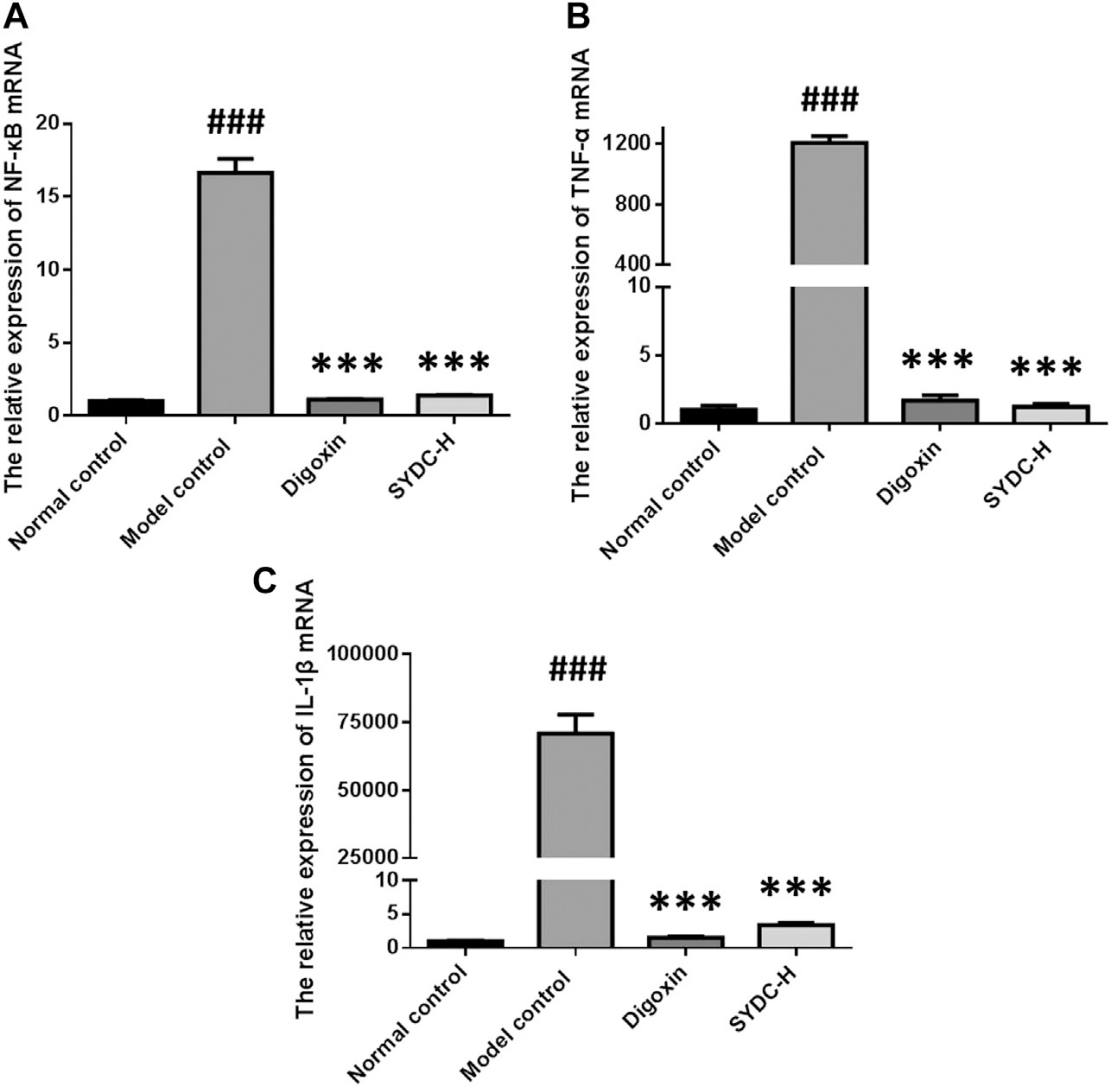
The mRNA relative expression of NF-κB, TNF-α, and IL-1β of HF zebrafish treated with SYDC for 4 h. ^###^Compared with the normal control, *p* < 0.001; ***compared with the model control, *p* < 0.001.

As shown in Figure 6, the mRNA relative expressions of NF-κB, TNF-α, and IL-1β of HF zebrafish in the model control group were significantly increased compared with those of HF zebrafish in the normal control group (16.65 ± 0.95 *versus* 1.00 ± 0.08, *p* < 0.001; 1,204.50 ± 46.78 *versus* 1.00 ± 0.30, *p* < 0.001; 70,859.65 ± 6,903.64 *versus* 1.00 ± 0.08, *p* < 0.001). The mRNA relative expressions of NF-κB of HF zebrafish in the SYDC-H treatment group and digoxin treatment group were significantly decreased compared with those of HF zebrafish in the model control group (1.39 ± 0.03 *versus* 16.65 ± 0.95, *p* < 0.001; 1.10 ± 0.05 *versus* 16.65 ± 0.95, *p* < 0.001). The mRNA relative expressions of TNF-α of HF zebrafish in the SYDC-H treatment group and digoxin treatment group were significantly decreased compared with those of HF zebrafish in the model control group (1.21 ± 0.22 *versus* 1,204.50 ± 46.78, *p* < 0.001; 1.68 ± 0.39 *versus* 1,204.50 ± 46.78, *p* < 0.01). The mRNA relative expressions of IL-1β of HF zebrafish in the SYDC-H treatment group and digoxin treatment group were significantly decreased compared with those of HF zebrafish in the model control group (3.38 ± 0.32 *versus* 70,859.65 ± 6,903.64, *p* < 0.001; 1.49 ± 0.18 *versus* 70,859.65 ± 6,903.64, *p* < 0.001). There were no significant differences in mRNA relative expressions of TNF-α, NF-κB, and IL-1β between the two drug treatment groups (*p* > 0.05).

### SYDC Inhibits Apoptosis in HF Zebrafish

**FIGURE 7:**
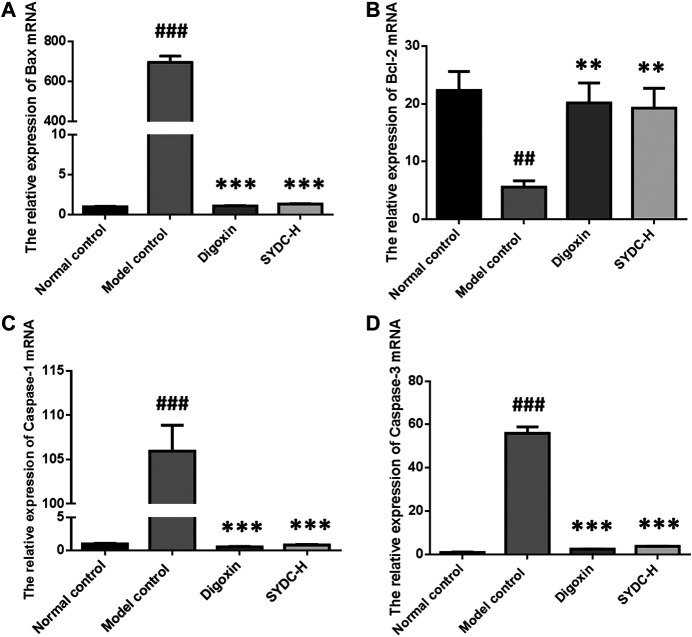
The mRNA relative expression of Bax, Bcl-2, Caspase-1, and Caspase-3 of HF zebrafish treated with SYDC for 4 h. ^###^Compared with the normal control, *p* < 0.001; **compared with the model control, *p* < 0.01; ***Compared with the model control, *p* < 0.001.

As shown in Figure 7, the mRNA relative expression of Bax of HF zebrafish in the model control group was significantly increased compared with that of HF zebrafish in the normal control group (695.36 ± 31.39 *versus* 1.00 ± 0.05, *p* < 0.001). In contrast, the mRNA relative expression of Bcl-2 of HF zebrafish in the model control group was significantly decreased compared with that of HF zebrafish in the normal control group (23.48 ± 4.89 *versus* 4.92 ± 1.58, *p* < 0.001). The relative mRNA expressions of Bax of HF zebrafish in the SYDC-H treatment group and digoxin treatment group were significantly decreased compared with those of HF zebrafish in the model control group (1.34 ± 0.03 *versus* 695.36 ± 31.39; 1.09 ± 0.06 *versus* 695.36 ± 31.39, *p* < 0.001). The relative mRNA expressions of Bcl-2 of HF zebrafish in the SYDC-H treatment group and digoxin treatment group were significantly changed compared with those of HF zebrafish in the model control group (18.79 ± 4.68 *versus* 4.92 ± 1.58; 20.56 ± 4.08 *versus* 4.92 ± 1.58, *p* < 0.01).

The mRNA relative expression of Caspase-1 and Caspase-3 of HF zebrafish in the model control group was significantly increased compared with that of HF zebrafish in the normal control group (105.95 ± 2.93 *versus* 1.00 ± 0.10; 54.23 ± 5.24 *versus* 0.65 ± 0.15, *p* < 0.001). The relative mRNA expressions of Caspase-1 of HF zebrafish in SYDC-H treatment group and digoxin treatment group were significantly decreased compared with those of HF zebrafish in the model control group (0.85 ± 0.06 *versus* 105.95 ± 2.93; 0.56 ± 0.07 *versus* 105.95 ± 2.93, *p* < 0.001). The relative mRNA expressions of Caspase-3 of HF zebrafish in SYDC-H treatment group and digoxin treatment group were significantly decreased compared with those of HF zebrafish in the model control group (4.78 ± 0.06 *versus* 54.23 ± 5.24; 3.27 ± 0.16 *versus* 54.23 ± 5.24, *p* < 0.001).

## Discussion

HF is the end stage of different ischemic cardiovascular diseases, including unstable angina pectoris and myocardial infarction (Burcu et al., 2013). Inflammation and apoptosis induced by oxidative stress have crucial roles in the occurrence and development of HF. SYDC, a hospital preparation of traditional Chinese herbal medicine, can prevent ischemic cardiovascular diseases through its antioxidative and anti-inflammatory effects (Liu et al., 2013; Zhou et al., 2017). In the present study, we demonstrated that SYDC attenuated verapamil-induced HF zebrafish. The relevant mechanism may be related to its anti-inflammatory and antiapoptotic effects via inhibiting the ROS-mediated NF-κB pathway.

The commonly used mammalian *in vivo* HF model is usually laborious, costly, and time-consuming, which restricts their application in pharmacology (Delgad, 2004). In recent years, zebrafish, a novel cardiovascular system animal model with good visibility, has been used for assessing drug toxicity, efficacy, and drug screening (Huang et al., 2013; Kim et al., 2013; Shi et al., 2017). Zhu *et al.* reported that the verapamil-treated larval zebrafish was convenient and predictive for rapid *in vivo* efficacy assessment and screening of HF therapeutic drugs because it developed pericardial edema and venous blood congestion with reduced cardiac output and blood flow velocity in larval zebrafish, similar to the pathophysiology observed in HF patients (Zhu et al., 2018). Our results showed that, compared with the normal control group, zebrafish treated with verapamil had obvious HF phenotypes such as cardiac enlargement and venous congestion. Compared with the normal control group, the heart output, blood flow velocity, and heart rate of zebrafish in the model control group were significantly increased compared with those in the normal control group. These data suggested that the HF zebrafish model was successfully reproduced and that it can be used as a repeatable, replaceable, simple, and novel experimental animal model of HF.

Clinically, SYDC, as one of our hospital preparations, has been used for clinical treatment of unstable angina pectoris for more than 30 years with favorable therapeutic effects (Li et al., 2019). SYDC is a TCM compound containing eight traditional herbs: *Hirudo*, *Astragali Radix*, *Codonopsis Radix*, *Pheretima*, *Eupolyphaga Seu Steleophaga*, *Corydalis Rhizoma*, *Salviae Miltiorrhizae Radix et Rhizoma*, and *Scrophulariae Radix*. Studies have shown that SYDC improved the clinical symptoms in patients with unstable angina (Li et al., 2019) and exerted myocardial protective effect through endothelial protection, antioxidative stress, and other mechanisms (Liu et al., 2013). Because zebrafish are sensitive to the effect of drugs, some adverse reactions, such as yolk sac blackening and heart rate slowing, may occur. Therefore, before the beginning of the efficacy study of SYDC on HF zebrafish, we firstly determined the maximum tolerance concentration (MTC) of SYDC for zebrafish. Our data suggested that zebrafish can tolerate SYDC that had good safety when administered at concentrations below 2,000 μg/ml. Further, our data also suggested that SYDC had a significant improving effect on HF zebrafish model by reducing areas of an enlarged heart and venous congestion and by increasing cardiac output, blood flow velocity, and heart rate. Moreover, this improving effect of SYDC on HF zebrafish was obviously dose-dependent.

Next, we investigated the mechanisms of SYDC attenuating HF zebrafish. In the development of HF, oxidative stress, inflammation, and apoptosis were shown to interact with each other. Oxidative stress is thought to be involved in inflammation. ROS accumulation induced by oxidative stress is considered a key molecule that activates the inflammatory signaling pathway. On the other hand, the inflammation response could generate more ROS that will aggravate oxidative stress (D’Aiuto et al., 2010; Fernández-Sánchez et al., 2011; Bondia-Pons et al., 2012). Our previous study showed that SYDC could inhibit oxygen-free radicals in ischemic myocardium (Liu et al., 2013). In the present study, we also demonstrated that SYDC could significantly inhibit excessive oxidative stress by reducing the ROS production and MDA levels and increasing the levels of SOD in HF zebrafish. This suggests that SYDC may have a cardioprotective effect by inhibiting excessive oxidative stress in different animal disease models. Our previous study showed that SYDC could inhibit the mRNA expression of TNF-α and NF-κB in the aorta of atherosclerotic mice. Our experimental data showed that SYDC could inhibit inflammation reaction by reducing the mRNA expressions of TNF-α, NF-κB, and IL-1β in HF zebrafish. ROS is a key molecule that activates the inflammatory signaling pathway, such as the NF-κB pathway, which is also a major regulator of the oxidative stress response (Savita et al., 2009; Bondia-Pons et al., 2012). Taken together, we speculate that SYDC may inhibit TNF-α and IL-1β by inhibiting the ROS-induced NF-κB signaling pathway in the verapamil-treated larval zebrafish model.

NF-κB is actually regarded as the matchmaker between apoptosis and inflammation (Galluzzi et al., 2011). NF-κB can transactivate genes with antiapoptotic functions, such as BCL-2, or lead to the production of proinflammatory mediators, including TNF-α (Perkins, 2008). Moreover, ROS-induced oxidative stress activates apoptosis pathways by upregulating Bax protein and caspase enzyme and downregulating the Bcl-2 (Stanely and Hemalatha, 2018). Bax, Caspase-3, and Bcl-2 are the endogenous mitochondrial apoptosis-related proteins. Caspase-3 is the most important executor of apoptosis, while Caspase-1 is the main inflammatory modulator in the caspase family. Activating Caspase-1 can induce the release of the level of IL-1β and IL-18. IL-18 can promote the production of TNF-α, while TNF-α reacts to Caspase-1, thus forming an inflammation reaction waterfall, promoting cell death, and eventually leading to HF (Mieczysław et al., 2020). In the present study, we also demonstrated that SYDC could inhibit apoptosis by downregulating the mRNA expressions of Bax, Caspase-3, and Caspase-1 and upregulating the mRNA expressions of Bcl-2 in the verapamil-treated larval zebrafish model. Moreover, we speculate that the antiapoptotic effects of SYDC may be related to the inhibiting effect of the ROS-induced NF-κB signaling pathway in the verapamil-treated larval zebrafish model.

In conclusion, SYDC attenuates verapamil-induced HF zebrafish and exerted anti-inflammatory and antiapoptotic effects. The inhibiting effects of SYDC on the ROS-mediated NF-κB pathway may be the key points in its crosstalk mechanisms between anti-inflammatory and antiapoptotic effects.

## Data Availability

The original contributions presented in the study are included in the article/Supplementary Material; further inquiries can be directed to the corresponding author.
