# Supplementary figures and images for: Shen-Yuan-Dan Capsule Attenuates Verapamil-Induced Zebrafish Heart Failure and Exerts Antiapoptotic and Anti-Inflammatory Effects via Reactive Oxygen Species–Induced NF-κB Pathway
Source: Front Pharmacol. 2021 Mar 1;12:626515. doi: 10.3389/fphar.2021.626515 (PMC7959770; doi:10.3389/fphar.2021.626515)

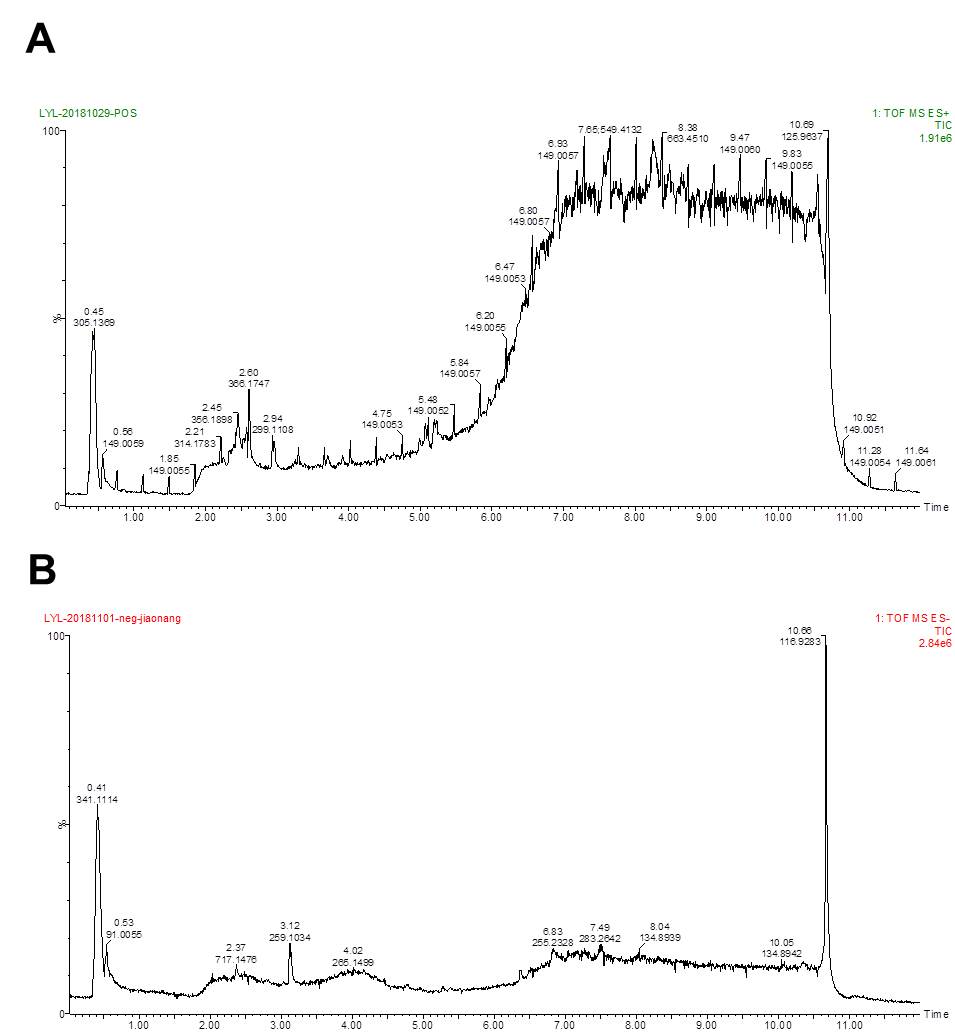

Supplement: Supplementary file 1 [file image1.jpeg]

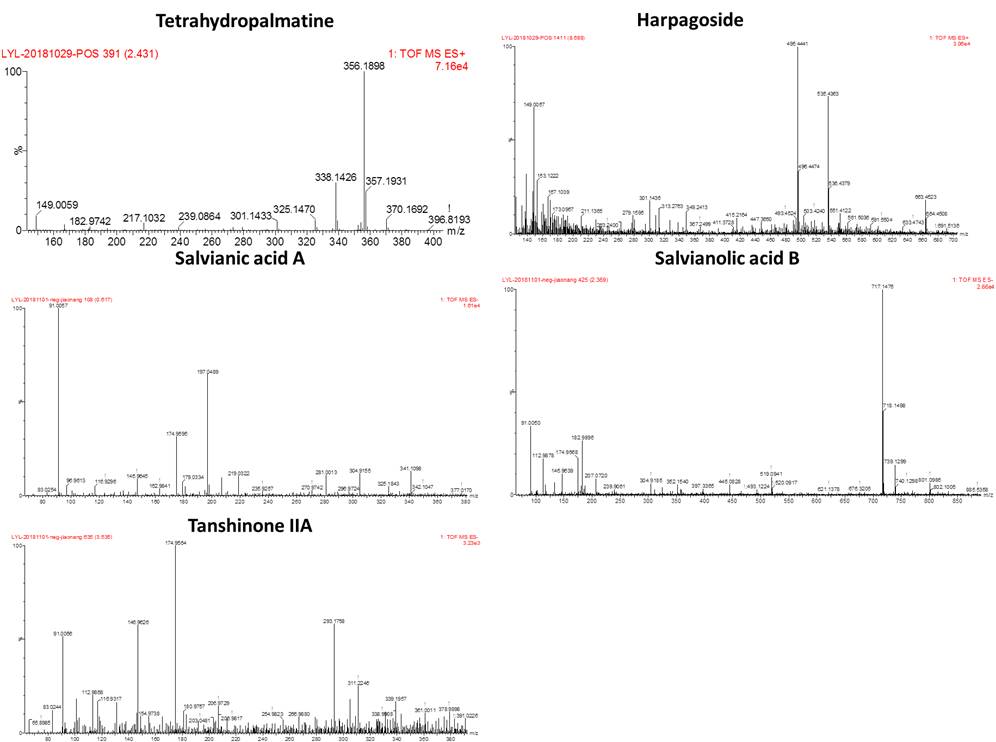

Supplement: Supplementary file 2 [file image2.jpeg]

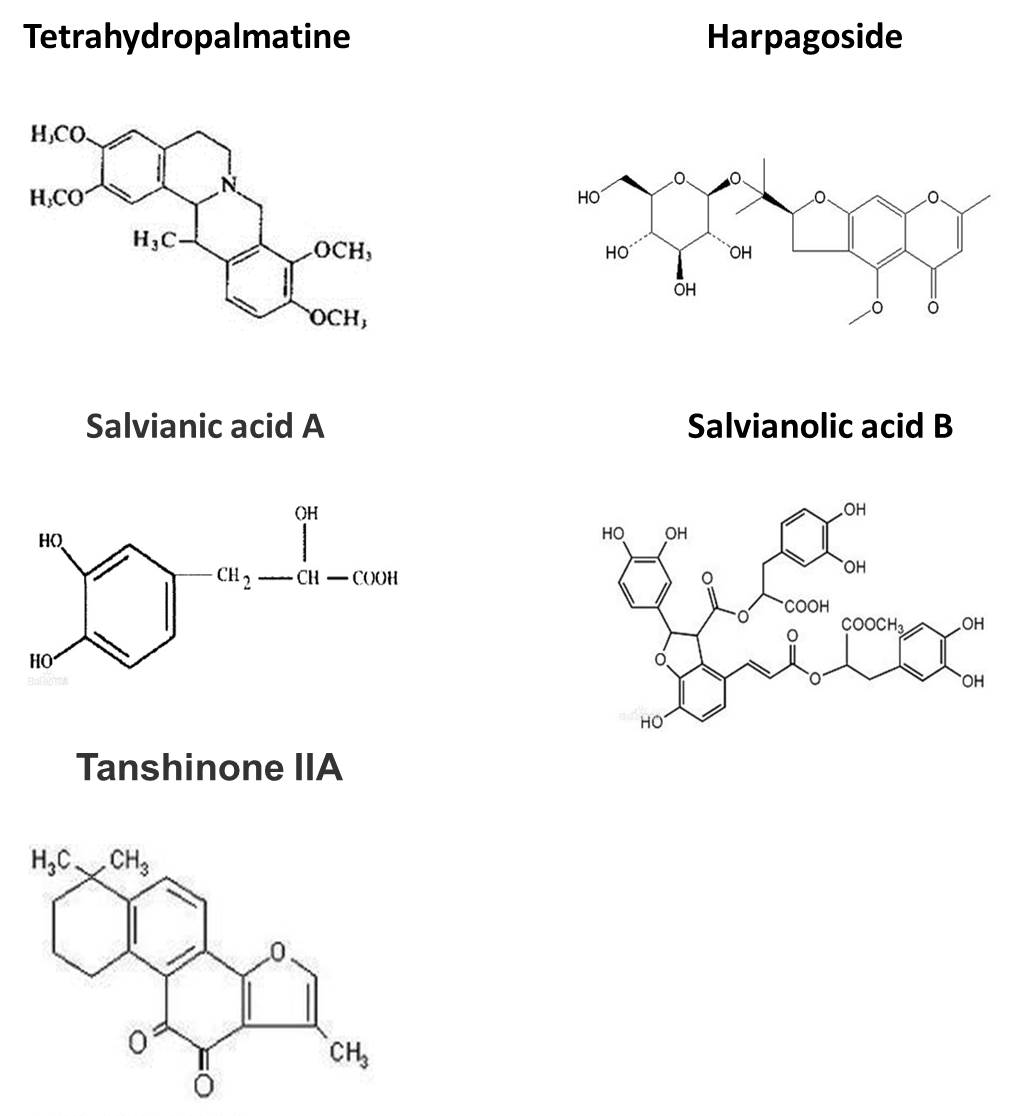

Supplement: Supplementary file 3 [file image3.jpeg]
